# Smaller Bioprosthetic Valves May Be Associated with Worse Clinical Outcomes and Reduced Freedom from Reoperation in sAVR

**DOI:** 10.3390/jcdd12070277

**Published:** 2025-07-18

**Authors:** Oliver Lee, David Derish, Dominique Shum-Tim

**Affiliations:** 1 UCL Medical School, University College London, London WC1E 6BT, UK; o.lee.20@ucl.ac.uk; 2 McGill University Health Centre, Division of Cardiac Surgery, Department of Surgery, McGill University Faculty of Medicine, Montreal, QC H3A 1A3, Canada; david.derish@mail.mcgill.ca

**Keywords:** valve size, bioprosthesis, surgical aortic valve replacement, clinical outcomes

## Abstract

Background: Surgical bioprosthetic aortic valve replacement is a ubiquitous procedure, with several factors identified in affecting outcomes. We hypothesize that smaller valves may be associated with worse outcomes and decreased freedom from clinical events, and a shift in implanting larger valves whenever possible may confer benefit to the patient. Methods: A narrative review of the literature was conducted using a systematic search strategy to evaluate studies examining the relationship between bioprosthetic valve size and outcomes. Inclusion criteria focused on studies reporting paired data on valve size and clinical endpoints in surgical AVR. Results: Among the 15 reviewed studies, smaller valve sizes were consistently associated with higher post-operative transvalvular gradients (6/7 studies) and increased reintervention rates (5/8 studies). Associations with accelerated structural valve degeneration (SVD) (3/5 studies) and reduced survival (8/11 studies) were also observed, although heterogeneity in study design and follow-up durations limited definitive conclusions. Conclusion: Our findings suggest that larger valve sizes may improve freedom from SVD, reduce reintervention rates, and enhanced survival. This may also justify the slight increased risk of enlarging the aortic root to accommodate a larger bioprosthetic valve prosthesis. Further high-quality, controlled studies are needed to clarify the independent impact of valve size on long-term outcomes and guide surgical decision-making.

## 1. Introduction

Aortic valvular disease poses a significant global health burden, with infectious etiologies like rheumatic fever dominating in low-income countries, while degenerative calcific disease prevails in high-income nations [1]. There are different options for the treatment of valvular disease, depending on several patient factors. Mild to moderate valvular disease, determined by symptom profile, patient function, and echocardiographic findings may be medically managed through antihypertensives and diuresis. However, as disease progresses and cardiac function declines, interventional management such as surgery or transcatheter replacement is required. While current contemporary trends towards the surgical treatment of aortic valve disease have moved towards a transcatheter approach, surgical approaches withstand the test of time, remaining the gold standard due to complicated patient anatomy, e.g., small aortic annulus, and concomitant procedures among other reasons.

Traditionally, mechanical valves were primarily recommended to younger patients less than 60–65 years old as mechanical valves were associated with increased durability [2]. However, they are more thrombogenic and require life-long anticoagulation usually in the form of warfarin, as well as constant monitoring and titration of therapeutic dose. This may negatively affect patient quality of life, particularly in active recipients. In older patients, bioprosthesis is more commonly recommended. This is due to bioprosthesis not requiring regular anticoagulation with warfarin and vitamin K antagonists [3] and therefore reducing the risk of haemorrhagic events in the elderly population. Additionally, in contrast with mechanical valves, bioprosthesis have finite durability, which is often in direct relation to the age of the recipient, and thus implying potential need for future reoperation in younger patients. However, recent advancements in the durability of bioprostheses and the efficacy of transcatheter valve-in-valve reoperation procedures have propelled bioprosthesis into a more favorable choice. As a result, the pendulum of prosthetic choice has shifted tremendously over the past decade. Younger patients now may wish to avoid the lifestyle restrictions, frequent medications and INR monitoring that comes with mechanical prosthetics, and so thus opt towards bioprosthetic heart valves.

Despite the growing use of bioprosthetic valves, controversy remains regarding the impact of valve size on haemodynamics, durability, and survival. Larger valve sizes are generally preferred to reduce transvalvular gradients and improve transcatheter valve-in-valve feasibility [4]. Some studies suggest better survival and lower rates of structural valve degeneration with larger valves, while other studies purport that there is no link and that valve design plays a more significant role.

We hypothesize that larger valves are associated with better freedom from structural valvular degeneration, prolonged survival and reduced rates of reoperation. This review aims to review the literature on bioprosthetic valve size and associated clinical outcomes.

## 2. Materials and Methods

Significant variations in the data used to investigate valve size effect made a systematic review difficult to perform. A narrative review approach was thus chosen. There was significant heterogeneity within patient populations and study characteristics. Distinctly, there were varying follow-up periods (7 months to 15 years), mixed valve types and models within studies, and an inconsistent patient population (Aortic stenosis and insufficiency). Additionally, many studies did not provide sufficient quantitative data for meta-analysis, lacking pairing between valve size and patient outcomes.

A systematic search method of returning viable literature was performed in order to maximize the sensitivity of the search strategy and thus the number of studies available for review. Electronic searches were performed using PubMed and Google Scholar. The following search terms used and combined were as follows:

- “Aortic Valve Replacement” “[MeSH]”;
- “Bioprosthetic SAVR”;
- “Prosthesis Size” OR “Valve Size” OR “Aortic Valve Prosthesis Size”.

Clear inclusion and exclusion criteria were defined to rule out non-viable studies that would not lend themselves to review. They are as follows:

Inclusion criteria:

- Published in English;
- Provides paired data or analysis on the relationship between valve size and outcomes of interest;
- At least one study group only performing surgical aortic valve replacement;
- Majority usage of bioprosthetic valves—porcine, pericardial, bovine, etc.

Exclusion criteria:

- Published in languages other than English;
- Provides data on valve size but not linked to outcomes;
- Does not provide data on specific valve sizes used;
- Only TAVR investigated;
- Only mechanical valves used;
- Published before 2000.

These criteria were designed so studies that simply included implanted valve size as a patient characteristic would be excluded, as without pairing to outcomes, conclusions could not be drawn. Furthermore, many papers investigating the impact of factors on post-sAVR outcomes comment on valve size, but only studies that include specific data on size implanted were included. Additionally, this review targets the effect of valve size on surgically implanted bioprosthesis only. Mechanical valve sizes and transcatheter implantation are not within the scope of this review and, as such, studies exclusively utilizing these parameters have been excluded.

## 3. Results

While a narrative format was ultimately selected, data collection was initially performed in order to establish the feasibility of a systematic review. Study characteristics such as design, sample size, valve size, and mean length of follow-up were collated and tabulated below in Table 1. A total of 15 studies fulfilled the inclusion criteria and were collected for review [5,6,7,8,9,10,11,12,13,14,15,16,17,18,19].

Any stated effect in aforementioned studies of implanted valve size on outcomes such as post-operative valvular gradients, reintervention rates, valvular degeneration, and mortality were collated and tabulated below in Table 2.

While studies might have commented on outcome variables of interest, if valve size was not mentioned as a statistically significant predictor or if an indirect relationship was not observed, it was stated as “Not recorded”.

Six out of seven studies found that smaller valves were significantly more prone to higher postoperative transvalvular gradients. Five out of eight studies found valve size to be directly linked to higher rates of reintervention. Three out of five studies found smaller valves to be more prone to earlier degeneration. Eight out of eleven studies found recipients of smaller valves to have reduced survival.

## 4. Discussion

### 4.1. Valve Sizing and Assessment

Prior to discussion of the influence of size on clinical outcomes, an understanding of valve sizing and how cardiac surgeons determine the optimal size of bioprosthesis is imperative. Appropriate sizing is crucial when considering echocardiographic and clinical outcomes of the patient. A prosthesis that does not adequately reduce transvalvular gradients will fail to reduce left ventricular afterload and will exacerbate left ventricular hypertrophy and heart failure. Additionally, poorly sized valves will increase rates of complications such as paravalvular leakage, structural valvular deterioration, pannus formation, and increased mortality [20,21].

Currently, pre-operative CT and echocardiography can be used to predict the dimensions of the patient’s aortic annulus, while the final valve size is performed intraoperatively using manufacturer provided apparatus. The surgeon then uses various sizing obturators, regarding the largest possible size as the optimal size for a given body surface area (BSA). Patients with particularly small aortic roots may necessitate a root enlargement procedure for the surgeon to implant an adequately sized prosthesis that confers favorable haemodynamic performance and mimics a native aortic valve [22,23]. However, enlargement does not come without risk. Factors such as surgeon experience and enlargement technique can influence outcomes, particularly the aortic cross-clamp time, which may increase perioperative morbidity and mortality [24].

Various specific metrics used to assess valvular fit and suitability. A key metric is prosthesis-patient mismatch (PPM), which has been shown to reliably predict higher transvalvular gradients [25] and thus indicate suitability of fit. Imaging modalities such as echocardiogram or CT is performed post-operatively to measure the effective orifice area offered by the implant. This is then divided by the patient’s BSA to calculate indexed EOA (iEOA). The required cardiac output of a patient is largely related to BSA, thus, if the EOA is not large enough to accommodate the increased flow in a larger patient, the patient will be subject to increased left ventricular afterload, preventing symptom relief and increasing the risk of SVD [26,27]. Pre-operative assessment of predicted EOA helps guide surgical strategies, including the decision to perform concomitant root enlargement procedures when severe PPM is anticipated. The degree of PPM is then categorized into mild, moderate or severe with moderate to severe degrees of PPM being highly unfavorable and linked to both early and late mortality, as well as reducing in left ventricular mass regression and CHF [25].

### 4.2. Impression

It is a well-established notion that high degrees of PPM lead directly to poorer clinical outcomes [25,28,29]. Quantifying the effect of valve size on the incidence of PPM with respect to individual patient factors is paramount to operative decision-making. It is widely agreed upon that there are certain factors that predispose patients to PPM such as BSA, valve size and advanced age [25]. Rates of PPM have been shown to be higher in patients receiving smaller valve sizes (<21 mm) [30]. As such, smaller patients who are more likely to have smaller valvular annuli [31] and receive smaller valves lend themselves towards PPM, and thus poorer clinical outcomes. Kolkailah et al. [10] found that the penalty on mortality exerted by PPM was partly dependent on the size of prosthesis received. Kolkailah et al. [10] observed that patients receiving a large prosthesis suffered more when exposed to the same degree of PPM as those with small prosthesis.

Feier et al. [7] contested the commonly held implications of PPM in smaller patients. While they affirmed that smaller valves were statistically more likely to exhibit mismatch, it failed to demonstrate an increase in 10-year mortality when propensity matched for comorbidities such as BSA, age, sex, and EuroSCORE II surgical risk score. Other papers that did not exclusively study bioprosthetic valves have demonstrated similar results in mixed and mechanical populations [28,29] suggesting that the current school of thought disparaging smaller valves may not be entirely true. These findings stress the importance of accurate calculation and interpretation of the projected iEOA as part of the pre-operative plan. Although it is possible to implant a >21 mm valve, it may be worth performing a root enlargement procedure in patients with larger BSA’s to avoid potentially severe mismatch. In fact, as operative expertise increases, root enlargement procedures have been shown to demonstrate excellent outcomes both intra-operatively with respect to cross-clamp time and post-operatively regarding mortality [23]. However, these findings should be considered within the context of the studies, as they contained mixed populations of biological and mechanical prosthesis, with no stratification between the two groups.

While biological prostheses do not require lifelong anticoagulation, conferring benefit to the patient’s quality of life and potential comorbidities, their durability is severely limited due to the inherent susceptibility to structural valvular degeneration (SVD). As the bioprosthesis was treated with glutaraldehyde to remove cellular debris and prevent immune rejection, cells lost the ability to regulate intracellular calcium, leading to a build-up of calcium phosphate (CaP) crystals [32]. In fact, the primary mechanism of failure in bovine pericardial valves is calcific structural valvular deterioration [33]. At the cellular level, bioprosthetic xenografts are commonly treated with glutaraldehyde. While strengthening the prosthesis through cross-linking and preventing rejection, cells lose the ability to regulate levels of intracellular calcium. This eventually leads to the build-up of calcium phosphate (CaP) crystals. Physiological calcium and phosphate present in blood is then able to support the growth of these calcium phosphate crystals into hydroxyapatite deposits, leading to calcification of the valve. Xenografts are also more prone to mechanically mediated degeneration due to the structural differences in the extracellular matrix [34]. The lack of fibrosa, spongiosa, and ventricularis layers reduce the tolerance of shear stress in biological valves. In addition, the glutaraldehyde treatment process cross-links collagen within the implants, restricting fiber motility. These factors work symbiotically, causing calcification to develop in areas of increased stress. As shear stress delaminates the fibrous component, the deposition of calcium on collagen and elastin fibers is accelerated and transvalvular gradients are then increased [35].

Once SVD of the bioprosthesis has occurred, management via redo aortic valve surgery or transcatheter valve-in-valve implantation is required [36]. The former is a suboptimal outcome due to the need to subject the patient to additional procedural risks as well as the additional trauma and recovery involved within an open redo procedure. Transcatheter valve-in-valve replacement demonstrates a low risk of all-cause mortality and intra-operative events, but is again not a desirable outcome, especially in younger patients.

Our review identified a mixed base of evidence for the association between valve size and incidence of SVD, with studies such as Salaun et al. [11] and Sénage et al. [17] supporting that small valve sizes were linked to earlier incidences of degeneration. Both studies found statistically significant differences in transvalvular gradients and rates of valvular degeneration in smaller valves. Larger valve sizes investigated in Sénage et al. [17] had significant lower gradients and lower rates of PPM. Sénage et al. and Salaun et al. [11,17] both corroborate the notion that smaller valves were associated with higher gradients and were predictors of SVD. Overall, Salaun et al. [11] reported hazard ratios of 2.1 (95% CI: 1.4–3.2) for structural valve deterioration in valves < 21 mm compared to larger sizes. Medallion et al. [6] found 15-year survival rates of 68% for 19 mm valves versus 78% for valves ≥ 25 mm. Johnston et al. [5] demonstrated mean gradients of 18.2 ± 8.4 mmHg for valves < 21 mm compared to 12.1 ± 5.6 mmHg for valves ≥ 25 mm at 5-year follow-up. Indeed, the main predictors of haemodynamic valvular deterioration identified in the studies were PPM, high trans-prosthetic gradients and regurgitation. Additionally, Salaun et al. [11] found that stentless bioprosthetics had weaker associations with deteriorations in haemodynamic performance due to SVD. This is likely due to the differences in design of stentless valves, providing less turbulence in flow and thus less mechanical strain on the leaflets [37]. This is explained and affirmed by Yun et al. [38] who demonstrated that stentless xenografts provided larger EOA’s and lower rates of PPM than stented bioprosthetics after 1 year. Salaun et al. [11] also points out that their study may be prone to survival bias, as patients who were unable to complete echocardiographic follow-up were excluded. Notably, a large proportion of these patients received size < 21 mm valves. The incidence of SVD in the study population may thus be higher than recorded.

However, other studies such as Danial et al. [12] found no link between size and incidence of SVD. Interestingly, the study population of Danial et al. [12] were exclusively patients with aortic insufficiency, and thus inherently had larger aortic roots [39]. They postulated that this could have been a contributing factor to the low rates of degeneration observed within their study. In fact, out of 289 patients undergoing sAVR for aortic insufficiency, only four incidences of SVD were reported, two of the cases receiving valves < 21 mm. In a separate paper, Johnston et al. [5] also found that valve size was an independent risk factor for survival, but not for reintervention. Rather, they observed prosthesis-patient mismatch and higher postoperative transvalvular gradients to have a stronger impact. This indirectly supports our hypothesis, as smaller valve sizes are associated with both PPM and higher gradients, leading to adverse outcomes. As reintervention is commonly used as a surrogate for SVD, which may skew the data as the decision to operate depends on a multitude of different factors such as haemodynamics, age, symptoms and operative risks. Patients in Johnston et al. may have also died before explant with undiagnosed SVD, further contributing to the potential underestimation.

Although outside the scope of this review, the impact of the valve model should also not be understated. Most studies included within this review included a variety of valve models implanted but did not investigate the impact that it had on outcomes. Thorough prediction of all factors influencing adverse outcomes is required to optimize patient care. Fuller et al. [14] demonstrates this while investigating outcomes in patients receiving transplantation for congenital heart disease. A variety of models were implanted, which found usage of the Sorin Mitroflow to be a predictor of re-intervention (yes that is very true, (probably trifecta valve also) but they are not recalcification but structural failure instead of degeneration for Sorin valve). Bajorek et al. [40] further emphasizes the importance of valve selection in a 5-year follow-up study comparing the effects on mortality in four different valves. The Hancock II valve was found to have significantly higher 5-year mortality, while the Trifecta valve was found to have the highest survival over the same period. Interestingly, the Abott Trifecta valves were later recalled by the FDA due to an earlier risk of SVD being identified [41], thought to be caused by the externally mounted leaflets in the valve contacting the surrounding aortic root and causing increased turbulence in flow, thus accelerating degeneration. These incongruous findings may be explained by shorter follow-up times and heterogeneity in the population, as the Hancock II population were significantly older with higher preoperative NYHA scores.

### 4.3. Technical Considerations Regarding PPM Assessment

Throughout this review, many studies made consistent references to PPM with regard to assessing valvular fit and suitability; however, the technical pitfalls and shortcomings of PPM should not be discounted. As previously mentioned, PPM is calculated through indexed effective orifice area (iEOA). Assessment of PPM through iEOA utilizing manufacturer measurements carries important technical limitations that must be acknowledged. Echocardiographic measurement of EOA using the continuity equation [42] is subject to several sources of error, including inaccurate left ventricular outflow tract (LVOT) diameter measurement, suboptimal Doppler alignment, and beat-to-beat variability. The assumption of circular LVOT geometry may introduce systematic errors, particularly in patients with elliptical outflow tracts. Furthermore, interobserver variability in EOA measurements can be substantial, potentially leading to misclassification of PPM severity. The flow-dependent nature of iEOA presents additional diagnostic challenges. In patients with reduced cardiac output or low-flow states, calculated iEOA may suggest PPM when valve function is actually normal (pseudo-PPM) [43]. Conversely, patients with hyperdynamic states may have apparently normal iEOA despite true PPM. This flow dependency necessitates careful clinical correlation and consideration of stroke volume index when interpreting PPM severity. Predicted iEOA can serve as a valuable preoperative planning tool. It allows surgeons to anticipate potential PPM before valve implantation and consider preventive strategies such as root enlargement. However, predicted values may not accurately reflect in vivo performance due to variations in implantation technique, patient-specific anatomy, and differences between in vitro testing conditions and physiological states.

Pressure recovery downstream of the valve should also be considered, particularly in patients with small aortic roots. In these cases, the pressure gradient measured immediately downstream may overestimate the true transvalvular gradient, as some pressure is recovered due to flow deceleration in the ascending aorta. This phenomenon is more pronounced with smaller valve sizes and may contribute to overestimation of PPM severity in certain patients [44].

## 5. Limitations

In order to substantiate a shift in clinical practice, more rigorous evidence is required behind the effect on valve size on clinical outcomes. There exists significant heterogeneity within the literature around the subject, and to our knowledge, a study focusing solely on the effect of valve size or an appropriate surrogate such as internal diameter does not exist. The confounding effect of external factors such as age, NYHA class or BSA makes it difficult to perform statistical analysis and make solid conclusions.

An important limitation of our review is the lack of standardized structural valve deterioration (SVD) definitions across included studies. Recent consensus studies from VARC-3 and EACTS [45] have established standardized SVD criteria, considering morphological features such as leaflet thickness and increased echogenecity, along with evidence of hemodynamic deterioration, to stage SVD. The studies included within this review spanned different time periods and employed different definitions. Some studies used echocardiographic criteria while others used reintervention and other composite endpoints. This heterogeneity in SVD definition may have influenced the reported associations between valve size and deterioration rates.

Additionally, studies varied in their approach to PPM assessment, with some focusing on absolute post-procedural gradients while others considered changes from baseline. This methodological inconsistency may have affected the strength of associations between valve size and hemodynamic outcomes, as baseline gradient consideration is crucial for accurate PPM evaluation.

The inclusion of studies with mixed patient populations (both aortic stenosis and aortic insufficiency) represents another limitation. While we included studies on aortic insufficiency patients to demonstrate outcomes in those receiving larger valve sizes, patients with aortic insufficiency typically have larger aortic roots and different hemodynamic profiles compared to those with stenosis, potentially confounding the relationship between valve size and outcomes. Future studies should stratify analyses by underlying pathology to better isolate the effect of valve size.

Additionally, follow-up time largely varies, ranging from 7 months to 15 years, making the ability to draw conclusions on the incidence of SVD and long-term mortality difficult. This is aggravated by the usage of surrogate endpoints in many studies, as the true incidence of outcomes such as SVD may be masked by asymptomaticity or death. Additionally, the studies mentioned utilize a range of different valve models and with some mixed populations, adding to the heterogeneity. Finally, publication bias within the field may also skew the findings, as clinicians are less likely to release non-significant or negative findings.

## 6. Recommendations

The nature of the subject matter makes randomized controlled trials logistically difficult to perform. Ethically, surgeons must select valve size and type that would firstly suit the patient’s anatomy with regard to their aortic root, but would also benefit the symptomaticity concerning ventricular function and cardiac output. As identified above, the heterogeneous nature of the data makes drawing conclusions difficult, thus, we recommend larger, well-controlled retrospective cohort studies that include paired data on valve size and model implanted and specific patient outcomes. This would not only enable more accurate isolation of the effect of valve size but would also allow the analysis of the effect with respect to valve size as well. This would hopefully facilitate the production of further level 1 evidence and improve patient outcomes in this increasingly important and ubiquitous procedure.

## 7. Conclusions

We initially hypothesized that larger valves are associated with improved clinical outcomes such as increased freedom from reoperation due to valvular degeneration, lower postoperative transvalvular gradients, and increased survival. Based on the literature reviewed, we believe that this hypothesis is true. Clinicians should carefully consider whether implanting a larger size would be feasible, especially in larger patients, and potentially consider root enlargement in patients with small aortic roots. This has the potential to provide lower transvalvular gradients, reduced incidence of PPM, and increased freedom from structural valvular degeneration. However, stronger levels of evidence regarding the association between valve size and clinical outcomes are required to substantiate a shift in operative practice.

## Figures and Tables

**Table 1:** Table detailing characteristics of studies that fulfilled the inclusion criteria.

Study Author	Country of Publication	Year of Publication	Study Size	Size Ranges	Average Time to Follow-Up (Years)
Johnston et al. [5]	USA	2024	2100	19–29	5.8
Medallion et al. [6]	USA	2000	892	19–33	5.0
Feier et al. [7]	Switzerland	2021	670	18–21	6.25
Yen et al. [8]	Japan	2024	695	19–27	NR
Kiaii et al. [9]	USA	2022	1118	17–29	5.0
Kolkailah et al. [10]	USA	2020	451	17–29	6.1
Salaun et al. [11]	USA	2018	1387	NR	6.2
Danial et al. [12]	France	2022	289	19–29	4.0
Gutfinger et al. [13]	USA	2024	23,197	NR	NR
Fuller et al. [14]	USA	2021	314	NR	2.9
Tadokoro et al. [15]	Japan	2021	474	19–27	1.3
Salna et al. [16]	USA	2017	2143	19–23	0.9
Sénage et al. [17]	USA	2014	617	19–27	3.8
Suvitesh et al. [18]	USA	2021	3444	NR	6.4
La Par et al. [19]	USA	2012	4621	19–31	NR

**Table 2:** Table of stated effect of implanted valve size on outcomes mentioned within studies.

Study Author	Transvalvular Gradient	Reintervention	Valvular Degeneration	Survival
Johnston et al. [5]	Smaller valves associated with higher gradients	Valve size not associated with reintervention; PPM and higher gradients associated with reintervention	Not specifically reported but reintervention deemed as surrogate	NR
Medallion et al. [6]	NR	NR	NR	Small valve sizes weakly associated with lower survival
Feier et al. [7]	Smaller valves more prone to PPM and thus higher gradients	Size not an independent risk factor for reintervention	NR	Small valve size group had lowest survival
Yen et al. [8]	Smaller sizes associated with higher gradients; PPM associated with higher gradients	Valve size not an independent factor for reintervention	NR	No association of valve size with survival
Kiaii et al. [9]	Smaller valves associated with increased gradients	NR	No cases of degeneration	NR
Kolkailah et al. [10]	NR	NR	NR	Severe PPM associated with reduced survival
Salaun et al. [11]	Smaller valves had higher post-operative gradients	Smaller valves linked with HVD incidence, requiring reintervention	Smaller valves and PPM more prone to degeneration	Smaller valves linked with higher HVD rates, deemed as predictor of survival
Danial et al. [12]	NR	No relationship described between valve size and reintervention	No relationship described between valve size and degeneration	No relationship described between valve size and survival
Gutfinger et al. [13]	NR	Smaller valves linked to higher rates of reintervention	NR	Valve size deemed as independent predictor of mortality
Fuller et al. [14]	NR	Smaller valve size linked to reintervention	NR	NR
Tadokoro et al. [15]	No relationship between valve size and mean gradients	NR	NR	NR
Salna et al. [16]	NR	NR	NR	Small valves associated with higher mortality
Sénage et al. [17]	Smaller valves had higher post-operative gradients	Small valves linked to earlier SVD, which may then necessitate reintervention	Small sizes linked to earlier SVD	Small valves linked to earlier SVD, which was linked to reduced survival
Suvitesh et al. [18]	NR	NR	NR	Larger sizes associated with increased survival
La Par et al. [19]	NR	NR	NR	Prosthesis size not associated with increased mortality

## Data Availability

No new data were generated or analyzed in support of this research. All data discussed are derived from previously published studies, which are cited within the manuscript.

## Abbreviations

The following abbreviations are used in this manuscript:

AI	Aortic Insufficiency
AVR	Aortic Valve Replacement
BSA	Body Surface Area
CaP	Calcium Phosphate
CHF	Congestive Heart Failure
EOA	Effective Orifice Area
HVD	Haemodynamic Valve Deterioration
INR	International Normalized Ratio
iEOA	Indexed Effective Orifice Area
NYHA	New York Heart Association
PPM	Patient-Prosthesis Mismatch
SAVR	Surgical Aortic Valve Replacement
TAVR	Transcatheter Aortic Valve Replacement
SVD	Structural Valve Deterioration
ViV	Valve-in-Valve (Procedure)
